# A COVID-19 Risk Assessment Decision Support System for General Practitioners: Design and Development Study

**DOI:** 10.2196/19786

**Published:** 2020-06-29

**Authors:** Ying Liu, Zhixiao Wang, Jingjing Ren, Yu Tian, Min Zhou, Tianshu Zhou, Kangli Ye, Yinghao Zhao, Yunqing Qiu, Jingsong Li

**Affiliations:** 1 The First Affiliated Hospital College of Medicine Zhejiang University Hangzhou China; 2 Engineering Research Center of EMR and Intelligent Expert System, Ministry of Education College of Biomedical Engineering and Instrument Science Zhejiang University Hangzhou China; 3 Research Center for Healthcare Data Science Zhejiang Lab Hangzhou China

**Keywords:** COVID-19, dynamic risk stratification, decision support system, telemedicine triage system, multiclass logistic regression

## Abstract

**Background:**

The coronavirus disease (COVID-19) has become an urgent and serious global public health crisis. Community engagement is the first line of defense in the fight against infectious diseases, and general practitioners (GPs) play an important role in it. GPs are facing unique challenges from disasters and pandemics in delivering health care. However, there is still no suitable mobile management system that can help GPs collect data, dynamically assess risks, and effectively triage or follow-up with patients with COVID-19.

**Objective:**

The aim of this study is to design, develop, and deploy a mobile-based decision support system for COVID-19 (DDC19) to assist GPs in collecting data, assessing risk, triaging, managing, and following up with patients during the COVID-19 outbreak.

**Methods:**

Based on the actual scenarios and the process of patients using health care, we analyzed the key issues that need to be solved and designed the main business flowchart of DDC19. We then constructed a COVID-19 dynamic risk stratification model with high recall and clinical interpretability, which was based on a multiclass logistic regression algorithm. Finally, through a 10-fold cross-validation to quantitatively evaluate the risk stratification ability of the model, a total of 2243 clinical data consisting of 36 dimension clinical features from fever clinics were used for training and evaluation of the model.

**Results:**

DDC19 is composed of three parts: mobile terminal apps for the patient-end and GP-end, and the database system. All mobile terminal devices were wirelessly connected to the back end data center to implement request sending and data transmission. We used low risk, moderate risk, and high risk as labels, and adopted a 10-fold cross-validation method to evaluate and test the COVID-19 dynamic risk stratification model in different scenarios (different dimensions of personal clinical data accessible at an earlier stage). The data set dimensions were (2243, 15) when only using the data of patients’ demographic information, clinical symptoms, and contact history; (2243, 35) when the results of blood tests were added; and (2243, 36) after obtaining the computed tomography imaging results of the patient. The average value of the three classification results of the macro–area under the curve were all above 0.71 in each scenario.

**Conclusions:**

DCC19 is a mobile decision support system designed and developed to assist GPs in providing dynamic risk assessments for patients with suspected COVID-19 during the outbreak, and the model had a good ability to predict risk levels in any scenario it covered.

## Introduction

### Background

In December 2019, cases of pneumonia with unknown cause, which was designated the coronavirus disease (COVID-19) in February by the World Health Organization (WHO), were reported in Wuhan, Hubei Province, China [1]. The number of cases of COVID-19 increased rapidly worldwide over the next few months, and the WHO announced a pandemic on March 11, 2020 [2]. As of April 29, 2020, 2,995,758 cases of infection and 204,987 deaths were reported worldwide [3]. COVID-19 has become an urgent and serious global public health crisis.

As a new infectious disease in humans caused by severe acute respiratory syndrome coronavirus 2 (SARS-COV-2), COVID-19 is characterized by respiratory symptoms and human-to-human transmission [4]. Rapid asymptomatic transmission of COVID-19 and high mortality in susceptible, elderly, and immunocompromised people make it necessary to identify and control positive patients quickly [5]. However, some characteristics of SARS-COV-2 have further increased the difficulty of early identification and isolation of patients. Wu et al’s [6] research showed that the mean basic reproduction number of SARS-CoV-2 is 2.68 (95% credible interval [CrI] 2.47-2.86) and that the epidemic doubling time is 6.4 days (95% CrI 5.8-7.1 days). Viral RNAs can be found in nasal discharge, sputum, and sometimes in blood or feces [7]. As described in past studies, similar to the other respiratory viral infections, the most common symptoms at the onset of COVID-19 were fever, cough, and fatigue [4,7], which increased difficulties in early identification of patients who were infected. Meanwhile, with the development of the epidemic, people’s panic and the shortage of medical resources has greatly hindered the prevention and control of the disease. In addition, research by Ji et al [8] revealed that the current mortality of COVID-19 is significantly positively related to the burden of health care. Therefore, how to evaluate patients with COVID-19, suspected cases, and other patients with similar symptoms, and effectively triage under the condition of medical resource shortages is of great significance to protect the susceptible population, reduce hospital cross infection, and decrease the burden on medical resources.

### Challenges to General Practitioners in the COVID-19 Outbreak

Community engagement is the first line of defense for effective prevention and control of infectious diseases [9]. In addition, in China, general practitioners (GPs) in primary care institutions play an important role in this battle. GPs are engaged in blocking the viral transmission by monitoring people at designated checkpoints, treating patients, and providing medical surveillance in the community. In addition, they detect, diagnose, and treat patients at different levels of fever clinics, providing continuous care for patients who are discharged and are chronically ill [9].

Disasters and pandemics pose unique challenges to health care delivery [10]. There are a lot of challenges for GPs and other health professionals to maintain their work and meet the increasing need for digital health care. First, the most serious problem is that they are at risk of getting infected; therefore, in-person visits need to be reduced to a minimum level. Second, many health service centers have experienced a rush of people who are worried or infected, and patients with chronic diseases who are undergoing treatment or observation are also at risk of being infected when they visit the outpatient clinics. Finally, medical information systems are generally interrupted and discontinuous; data still needs to be manually recorded or repeatedly registered by health professionals, which increases the workload of health professionals and makes it impossible to manage the dynamic information of patients in a timely and comprehensive manner.

### The Role of Telemedicine in the Era of COVID-19

Telemedicine has a critical role in emergency responses and is an ideal model for managing infectious diseases [10,11]. In addition, the key factor that slows the spread of the virus is the “social distance,” which can directly reduce person-to-person contact [11]. Telemedicine can also deploy large numbers of health care providers rapidly to facilitate triaging without in-person visits, provide clinical services when local medical resources are unable to meet the demand, and reduce the risk of nosocomial infection.

Until now, researchers have developed many different forms of telemedicine systems to meet the needs of fighting against the epidemic. Jin et al [12] built and deployed an artificial intelligence (AI)–assisted system for automatic computed tomography (CT) image analysis and recognition of COVID-19 within 4 weeks, which has been applied in many hospitals, greatly reducing the pressure of radiologists. Du et al [13] proposed a hybrid AI model for COVID-19 prediction that fully considered the effects of prevention and control measures, and the improvement of public prevention awareness, and has been applied in a number of Chinese cities. Moreover, Reeves et al [14] built a series of standardized tools based on the existing electronic health record (EHR) system for the COVID-19 epidemic to support outbreak management, including scripted triaging, electronic check-in, standard ordering and documentation, secure messaging, real time data analytics, and telemedicine capabilities.

These models and systems have considered the main problems from different perspectives during the COVID-19 epidemic and have been deployed and published in a short time, which has played a significant role in fighting the epidemic. However, to the best of our knowledge, there is still no suitable mobile management system that can help GPs realize the automatic collection of data, dynamic risk assessment, and effective triaging and follow-ups with patients with COVID-19, as well as the effective reduction of the pressure on large designated general hospitals.

### Study Aims

In this study, by integrating doctor experience, clinical guidelines, and retrospective data, we designed and developed a dynamic risk assessment decision support system for COVID-19 (DDC19) to assist GPs in data collection, dynamic risk assessment, triage management, and follow-ups during the outbreak of COVID-19. The DDC19 is designed to build a free mobile app that can cover all the different situations encountered by residents and GPs, and GPs can use it for dynamic continuity management. We describe our experiences, lessons learned, and recommendations for the design and implementation of telemedicine tools in future health emergencies.

## Methods

### The Covering Scenes of DDC19

To achieve the early assessment and triage of patients with COVID-19 and ease the pressure of shortages in medical resources, the following key issues need to be resolved: how to fully grasp and effectively manage the residents’ status in real time without increasing the GP's working burden and, without omitting potential patients with COVID-19, how to effectively use medical knowledge and risk stratification models to achieve effective evaluation and classification, as well as the patients’ scientific stratification.

**Figure 1:**
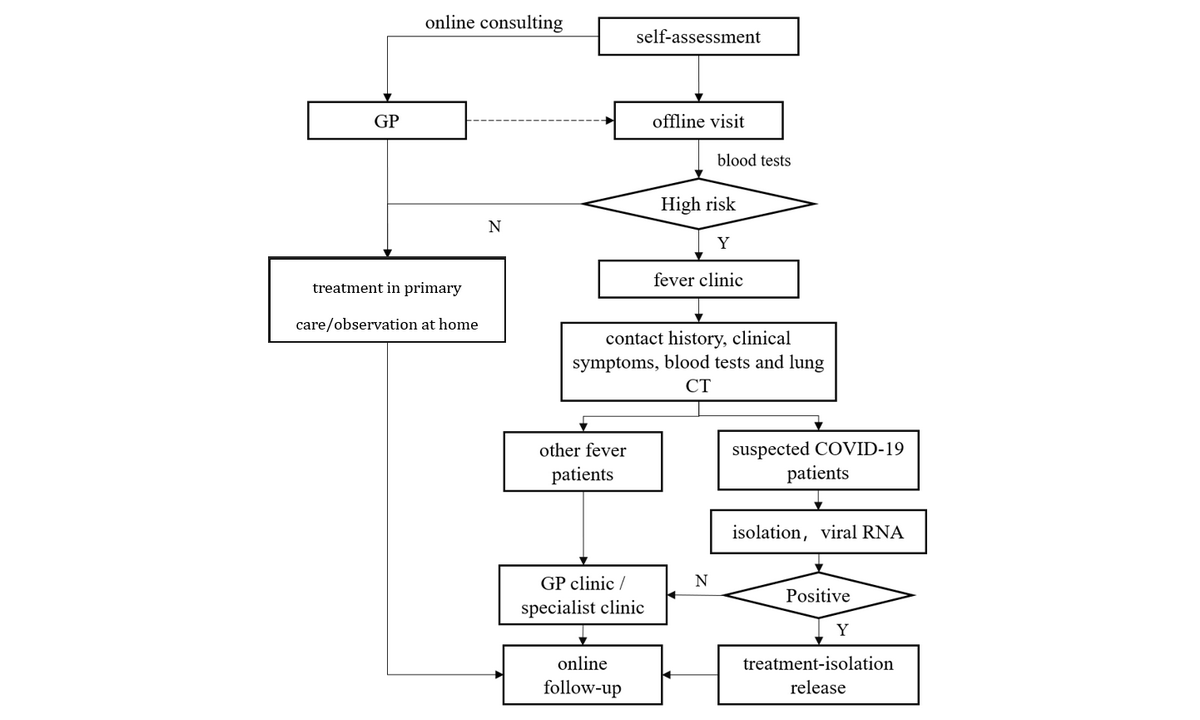
The covering scenes of patients using health care during the outbreak of COVID-19. COVID-19: coronavirus disease; CT: computed tomography; GP: general practitioner.

Accordingly, based on the principle of the four early approaches (early detection, early reporting, early isolation, and early treatment) and the actual scenes and process of patients using health care, DDC19 was designed to help GPs manage their patients who had a fever or respiratory symptoms, or suspected infection with SARS-CoV-2 during the outbreak of COVID-19. Several scenarios were involved and are demonstrated in Figure 1. First, patients should fill in the self-assessment questionnaire in the system. After that, according to the risk level assessed by the system and the diagnosis and treatment suggestions given, patients can choose to contact the GPs online for consultations or directly go to the primary care center for further examination and treatment. Second, when patients choose to visit in-person, those who are at low- and moderate-risk may receive blood tests and have their risks reassessed based on actual clinical symptoms and blood test results. After reassessment, if patients are still at low- and moderate-risk, GPs will give them some necessary treatment and follow-up online. For patients who are high risk, they should be transferred to a fever clinic as soon as possible. Third, patients at high risk who are transferred to the fever clinic will receive a lung CT scan and viral RNA test. If the lung CT shows a clinical manifestation of COVID-19 or the viral RNA test is positive, they will be isolated and treated. Otherwise, they will be transferred to the GP or specialist that is needed. Finally, the cured patients will return to the community, GPs will continue monitoring their conditions and dynamically assess risk online, as well as follow-up with them offline if needed.

### The Design of Main Functions and Business Process of DDC19

To make full use of the previously mentioned online-offline combined triage mode during the COVID-19 outbreak, we deeply integrated GP’s experience and suggestions, and designed the business process of the DDC19 system in detail. In the first step, we reviewed the COVID-19 guidelines [15] and fully integrated the actual clinical experience of GPs on the frontline of COVID-19 prevention and control to determine the main functions of the system and the content of the questionnaire. In the second step, based on the content of the questionnaire, the main data elements of the risk stratification model were determined, and, further considering the variability of the amount of the available questionnaire content data, a dynamic risk stratification model was constructed. In the third step, we validated and adjusted the model with retrospective clinical data. Therefore, the functions and business processes of the system were gradually completed according to the changes of clinical requirements.

**Figure 2:**
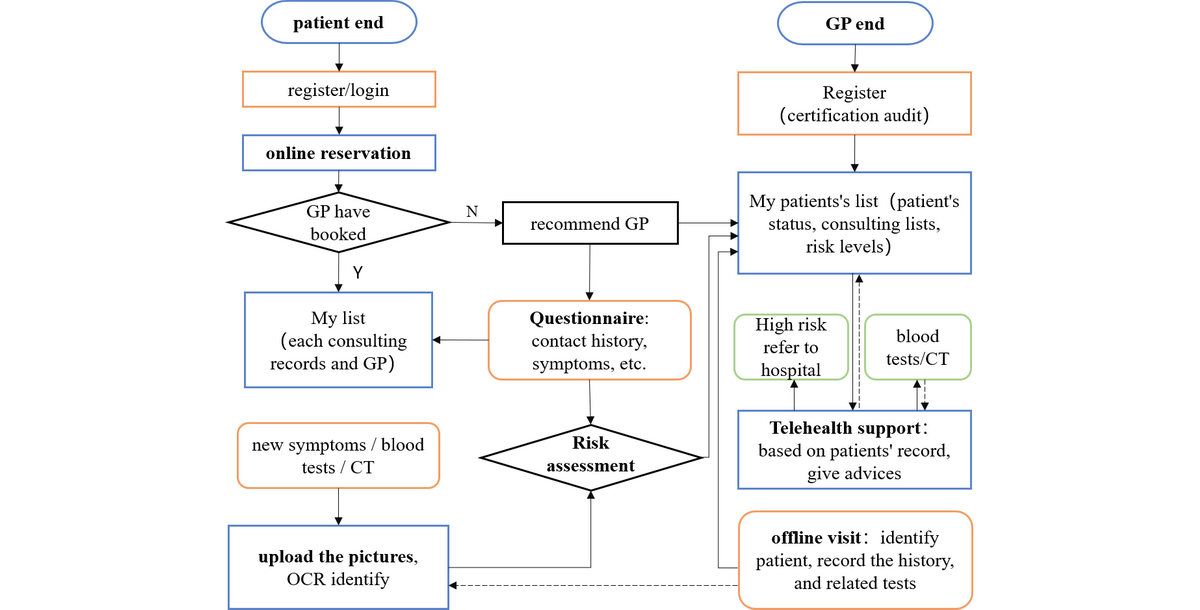
The main business flowchart of DDC19. CT: computed tomography; GP: general practitioner; OCR: optical character recognition.

As shown in Figure 2, the system mainly includes the patient-end and the doctor-end. Its main functions, including online patient clinical data collection, dynamic risk assessment, online classification, and appointment, can be achieved through the interaction between two mobile terminals. For the online collection module of the patient’s clinical data, it was completed by the structured questionnaires and uploading of examination report pictures. The questionnaire content related to the early assessment of COVID-19 will be gradually adjusted according to the experience of GPs and clinical guidelines [15]. The detailed information of the health questionnaire is shown in Multimedia Appendix 1. To ensure that patients with COVID-19 were not omitted and that enough attention was given to the patients with low risk and moderate risk, and to improve the reliability of risk assessment, a dynamic risk assessment module was designed based on the current clinical practice experience and expert recommendations, and the risk assessment level of COVID-19 was divided into low risk, moderate risk, and high risk. In addition, to ensure the patients’ risk level can be provided in real time and to consider the different dimensions of clinical data that patients uploaded, the dynamic risk stratification model, based on machine learning, was trained by the retrospective data (see details in the next section).

### The Construction of the COVID-19 Dynamic Risk Stratification Model

Considering the characteristics of COVID-19 such as unobvious symptom specificity and long incubation period, we constructed a COVID-19 dynamic risk stratification model with high recall and clinical interpretability. Based on a multiclass logistic regression algorithm, it integrates the retrospective clinical data analysis results of patients, doctors’ experiences, and clinical guidelines.

**Figure 3:**
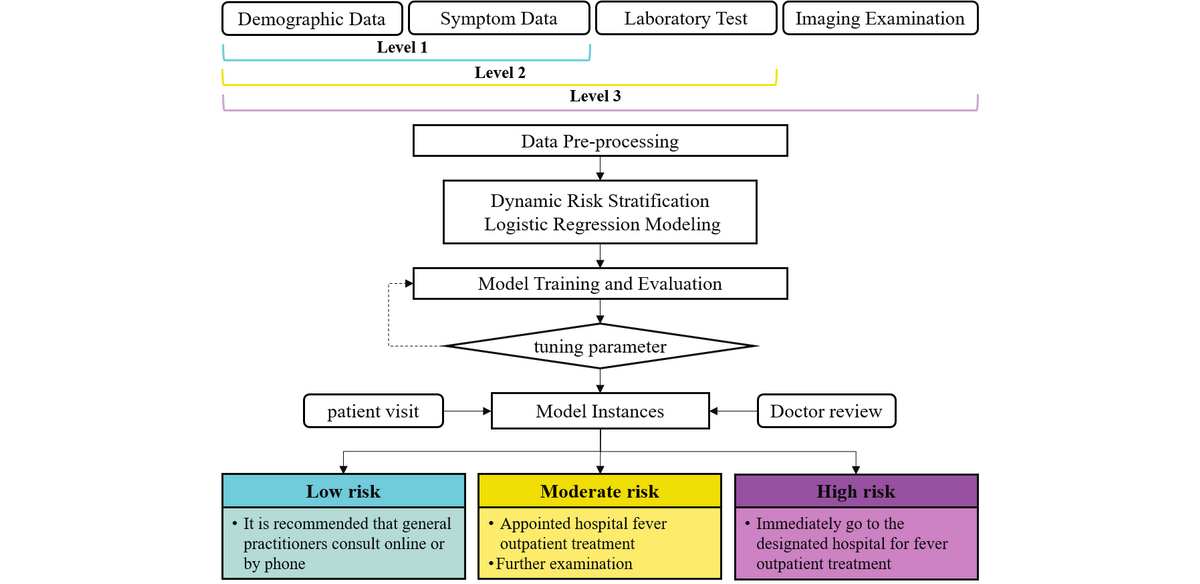
The construction process of the dynamic risk stratification model.

To meet the risk assessment requirements of COVID-19 at the system level for multiple levels and multiple scenarios, this model was based on patients’ data from the fever clinic during the COVID-19 epidemic period and patients’ risk levels provided by GPs according to COVID-19 diagnosis and treatment guidelines [15]. Currently, for patients who visit the fever clinic of designated hospitals during the epidemic, GPs will provide the COVID-19 risk assessment to them based on epidemiological history and clinical manifestations (symptoms, blood tests, and lung CT scan). The detailed content and process of evaluation are shown in Textbox 1. According to the different levels of health information submitted by patients in the system, the research team used the patient’s risk level (assessed by GPs according to clinical guidelines as patient’s label), demographic data, epidemiological history, clinical symptoms, data of laboratory tests, and lung CT imaging as the characteristics of the patient to construct a dynamic risk stratification model. The detailed model construction process is shown in Figure 3. Compared with the fixed-risk assessment method, which required all the information of patient’s medical history, laboratory examination, and the result of the lung CT, this model can not only provide COVID-19 risk stratification for new patients but also provide a dynamic risk level assessment and medical advice based on the clinical data submitted in the system by the patient at the different stages.

**The content and standards of coronavirus disease risk assessment.**

**Epidemiological history**

- Within 14 days before the onset of the disease, the patient has a travel or residence history in the high-risk regions or countries.
- Within 14 days before the onset of the disease, the patient has a history of contact with those infected with severe acute respiratory syndrome coronavirus 2 (those with a positive nucleic acid testing result).
- Within 14 days before the onset of the disease, the patient had direct contact with patients with fever or respiratory symptoms in high-risk regions or countries.
- Disease clustering (2 or more cases with fever or respiratory symptoms occur at such places as homes, offices, and school classrooms, within 2 weeks)

**Clinical manifestations**

- Fever or respiratory symptoms
- The white blood cell counts in the early stage of the disease is normal or decreased, or the lymphocyte count decreases over time.
- Computed tomography imaging features of the coronavirus disease

Table 1 shows the data elements used in the dynamic risk stratification model and their corresponding detailed descriptions. Data elements mainly include four categories: demographic data, epidemiological history, clinical symptoms (eg, fever, cough), the data of laboratory tests (eg, blood routine test and C-reactive protein [CRP] test), and lung CT imaging data. According to several studies [16-19], these data elements include the early clinical symptoms of COVID-19 and the easily accessible data of clinical examination, which plays an important role in early assessment.

**Table 1:** The data elements in the dynamic risk stratification model.

Main category, data element		Description	
**Patient demographic information**			
	Patient ID	Unique patient identifier	
	Gender	Patient’s gender identity	
	Age	Patient’s age	
**Clinical symptoms**			
	Fever	Normal: temprature≤37.2℃; low fever: temperature between 37.2℃ and 38.5℃; High fever: temperature≥38.5℃	
	Cough	Cough or dry cough	
	Sputum production	N/A^a^	
	Fatigue	N/A	
	Breathing	Shortness of breath, anhelation, polypnea, etc	
	Chest uncomfortable	Chest pain or chest distress	
	Pharyngalgia	Pharyngalgia	
	Headache	Headache or dizziness	
	Chills	Fear of cold	
	Soreness	Body aches, joint pain, myalgia	
	Stuffy nose	Stuffy nose or runny nose	
	Gastrointestinal reactions	Feeling sick, vomiting, abdominal pain, diarrhea, etc	
**Epidemiology history**			
	Contact history	Have a COVID-19^b^ contact history	
**Imaging examination**			
	CT^c^	Lung CT shows viral pneumonia	
**Blood routine examination**			
	WBC^d^	White blood cell count (10E9/L)	
	GRAN^e^	Neutrophil count (10E9/L)	
	LYM^f^	Lymphocyte count (10E9/L)	
	RBC^g^	Red blood cell count (10E12/L)	
	HGB^h^	Hemoglobin concentration (g/L)	
	HCT^i^	Hematocrit (%)	
	MCV^j^	Mean corpuscular volume (fl)	
	MCH^k^	Mean hemoglobin content (pg)	
	MCHC^l^	Mean corpuscular hemoglobin concentration (g/L)	
	RDW^m^	Red blood cell distribution width (%)	
	PLT^n^	Blood platelet count (10E9/L)	
	MPV^o^	Mean platelet volume (fl)	
	PCT^p^	Platelet hematocrit (%)	
	PDW^q^	Platelet distribution width (10 [GSD^r^])	
	MO^s^	Mononuclear cell count (10E9/L)	
	EO^t^	Eosinophil count (10E9/L)	
	BA^u^	Basophil count (10E9/L)	
	NRBC^v^	Percentage of nucleated red blood cells	
	IG^w^	Immature granulocyte percentage (%)	
	CRPH^x^	C-reactive protein (mg/L)	

^a^Not applicable.

^b^COVID-19: coronavirus disease.

^c^CT: computed tomography.

^d^WBC: white blood cell.

^e^GRAN: granulocytes.

^f^LYM: lymphocyte.

^g^RBC: red blood cell.

^h^HGB: hemoglobin.

^i^HCT: hematocrit.

^j^MCV: mean corpuscular volume.

^k^MHC: mean hemoglobin content.

^l^MCHC: mean corpuscular hemoglobin concentration.

^m^RDW: red blood cell distribution width.

^n^PLT: platelet.

^o^MPV: mean platelet volume.

^p^PCT: platelet hematocrit.

^q^PDW: platelet distribution width.

^r^GSD: geometric standard deviation.

^s^MO: mononuclear.

^t^EO: eosinophil.

^u^BA: basophil.

^v^NRBC: nucleated red blood cells.

^w^IG: immature granulocyte.

^x^CRPH: C-reactive protein.

After the optical character recognition (OCR) module and natural language processing (NLP) module recognize and preprocess the image and text data, the structured patient data is extracted from the app back end database to the data preprocessing module for subsequent data analysis; the detailed data elements are shown in Table 1. The system established a multiclass logistic regression model to dynamically predict and stratify the risk of COVID-19 infection based on the input of different levels of patient clinical data. Logistic regression has good classification ability and interpretability, which has been widely used in the field of machine learning. The hypothetical function is:


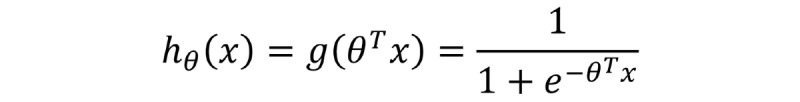


In this formula, *x* is the model input, and *θ* is the main parameter of the model. This parameter is obtained mainly by fitting the observed data features and corresponding labels. For the case of linear boundaries, *θ^T^x* can be explained as follows:


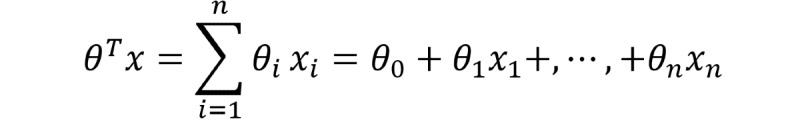


The output *h_θ_* (*X*) ∈ (0,1) represents the probability that the result is 1. Therefore, for the binary classification problem, the probability that the result of the input *x*classification is category 1 and category 0 is:

*P*(*y* = 1│*x*; *θ*) = *h_θ_* (*x*)

*P*(*y* = 0│*x*; *θ*) = 1 - *hθ* (*x*)

To measure the difference between the model prediction classification result *h_θ_* (*X*) and the true value *y*, the corresponding loss function is defined as:


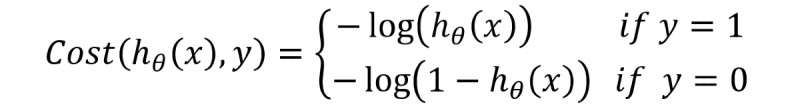


For m training samples, the loss function is expressed as follows:


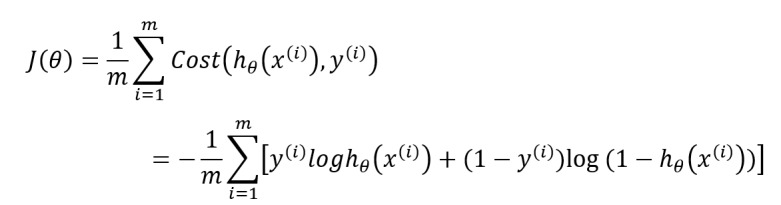


Based on the retrospective data of m patients, by minimizing the loss function *J*(*θ*), the parameter *θ* makes the predicted classification category closest to the patient's true risk level obtainable. However, to balance the distribution of medical resources as much as possible and allow more patients to get enough medical attention, the risk stratification is set to three categories: low risk, moderate risk, and high risk. Therefore, in terms of model construction, to adapt to the above multiclassification situation, the training set data is divided into two different categories to train multiple classifiers. Assuming that n represents the number of categories in the training set, then 
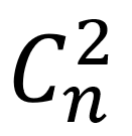
 classifiers need to be trained for prediction; the function is expressed as:


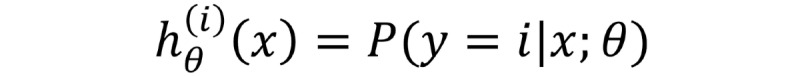


Where i is the i-th category. For the input data of the newly visited patient, 
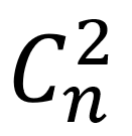
 classifiers will be used to predict the risk level, and the classification result with the highest output probability is taken as the risk level of the patient. The function is expressed as:


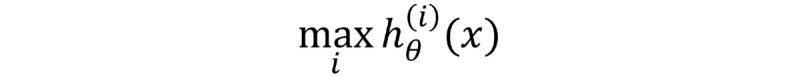


## Results

### The Architecture and Prototype of DDC19

To prove the effectiveness of the previously mentioned COVID-19 dynamic risk stratification model, as well as the feasibility and practicability of the early assessment and triage system, the research team developed the system prototype and put it into practical operation and application.

**Figure 4:**
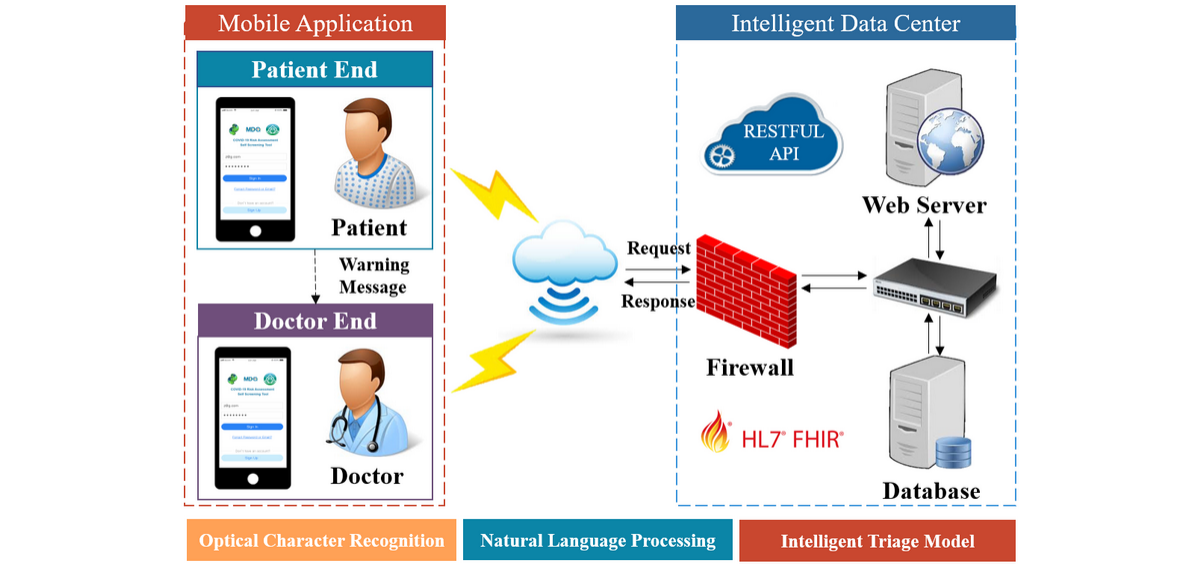
System architecture diagram. API: application programming interface; FHIR: Fast Healthcare Interoperability Resources; HL7: Health Level 7; RESTFUL: representational state transfer.

As shown in Figure 4, the DDC19 is mainly composed of three parts: two main mobile terminal apps (patient-end and GP-end), and the database system with its related components and underlying related support model. All mobile terminal devices connect to the back end data center wirelessly to achieve request sending and data transmission. In this section, the main architecture and functions of each part will be described in detail.

The mobile app end builds user interface based on the progressive Vue framework. The server end implements data and service interaction with the front end by the representational state transfer application programming interface specification. It is more concise and lighter, both for the processing of uniform resource locators and the encoding of the payload [20]. The server end builds a JavaScript scripting environment by Node.js; improves the system performance using event-driven, nonblocking, and asynchronous input and output models; and optimizes the transmission volume and scale of the app. The data storage adopts the MongoDB open-source database system, which has the characteristics of open-source, cross-platform, and powerful expansion capabilities. To achieve the storage and exchange of patient’s clinical data, structured processing data of the laboratory test, and image reports, the system adopts the standard framework of Fast Healthcare Interoperability Resources to ensure the accuracy of clinical data storage and expression. To make full use of the data in the form of pictures contained in the laboratory test and image reports, the system integrates the mature OCR module and NLP module to complete the identification and extraction of relevant clinical information in the picture.

**Figure 5:**
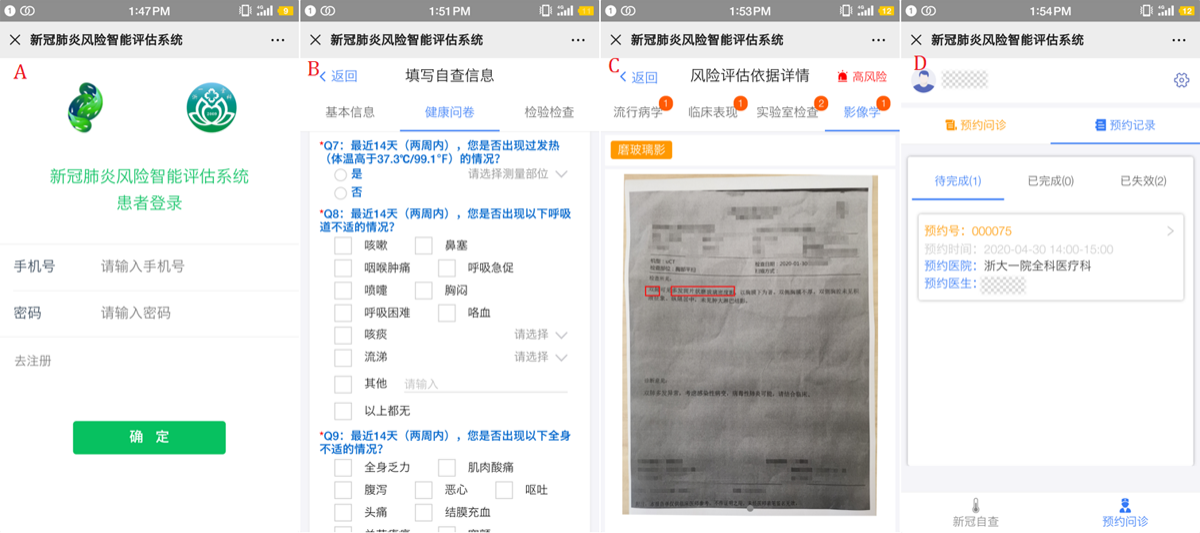
The screenshots of DDC19’s patient mobile terminal app.

For the patient-end (Figure 5A), the app assists patients in collecting, recording, and transferring their health data. There are two forms of recording health data for patients, including filling in a structured questionnaire by themselves and uploading pictures (Figure 5B and C). The range of health data includes the patient’s basic information, a health questionnaire related to COVID-19 early assessment, and clinical test results. In addition, the content of the health questionnaire will be gradually adjusted according to the relevant authoritative guidelines (see details in Multimedia Appendix 1) [15]. In addition, the data of clinical examination, including laboratory test reports such as blood routine test and CRP, and image examination reports such as lung CT reports can be uploaded as pictures directly by the camera of the mobile device. The mobile app communicates with the back end server through a message transmission mechanism. This mechanism establishes a balance between real time data transmission and system availability, which can completely meet the requirements of medical data transmission. After being analyzed by the back end database risk stratification model and related algorithms, the evaluation report will be returned to the patient app, mainly including results and evidence of the risk level evaluation and the corresponding detailed medical advice (Figure 5C). Patients can also update their symptoms and clinical data in real time on the app. The app will give new evaluation results and medical advice immediately. In addition, the patient app supports online patient appointments and provides a list of GPs working in the primary care clinics or fever clinics in designated hospitals (Figure 5D).

**Figure 6:**
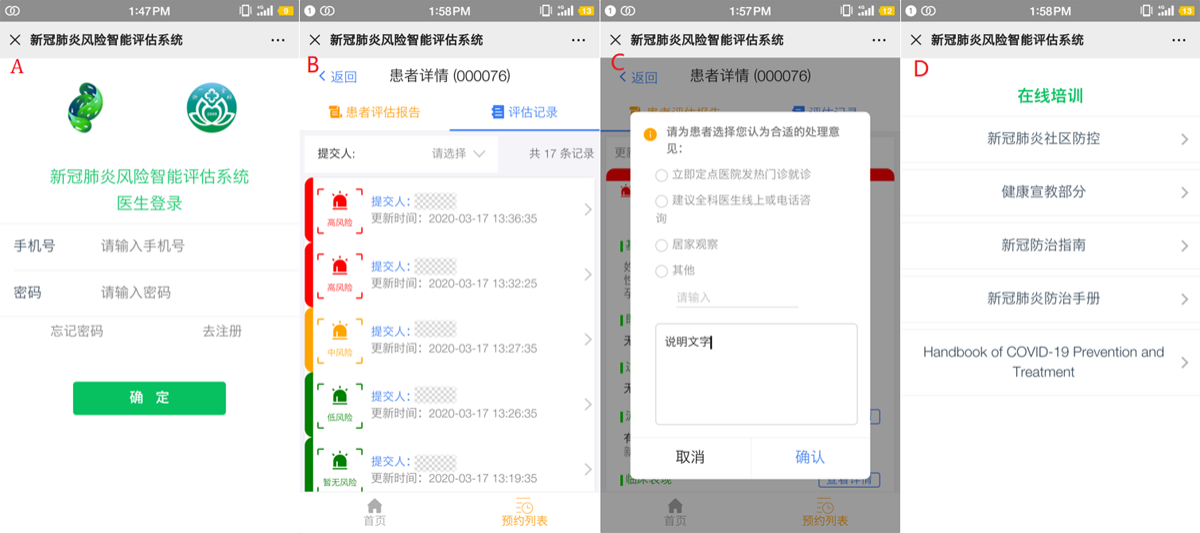
The screenshots of DDC19’s doctor mobile terminal app.

For the GP-end (Figure 6A), the app mainly assists GPs in managing their patients, including checking the information of their patients in the system, the results, and the basis of the automatic risk assessment, and giving recommendations based on the assessment results. First, it supports GPs to check their patients according to their risk level or appointment date and searches their patients by their name or mobile phone number (Figure 6B). Second, for the patient who has completed an in-person visit, it supports GPs in uploading the patient’s laboratory test results, image examination reports, and other data to the back end database to which the patient belongs, and they will obtain an update on the patient's risk assessment report. In addition, it also supports GPs in checking the details of their risk assessment results and the previous evaluation records, which helps GPs to grasp the patient's dynamic condition completely and in a timely manner. For a patient who is high risk that did not go to the designated hospital for further investigation timely, the system would send a reminder message for GPs to deal with it as soon as possible. At the same time, GPs can also give recommendations for the patient who applies for offline consultation based on their personal information and submitted clinical data (Figure 6C). GPs can grasp the status of all the residents they managed by these functions of the app. Third, to help GPs in different health institutions grasp the latest prevention and control knowledge of COVID-19 in real time, the GP-end has also developed the function of online training for GPs (Figure 6D).

### Case Studies and Data Validation

For the purpose of testing and verifying the dynamic risk stratification model of DDC19, 2243 patients’ information were collected from the fever clinic of the first affiliated hospital of Zhejiang University from January 19 to March 11, 2020. The data includes the patients’ basic information (gender and age), chief complaint, medical history, physical examination, laboratory tests, and the lung CT image examination reports. The first affiliated hospital of Zhejiang University is a class A hospital with 2500 beds. In 2019, the number of outpatient and emergency services reached 5 million and 243,300 were discharged.

The patient's medical record number is used as the unique identifier, and the data corresponding to the earliest outpatient visit record of each patient within the range from January 19, 2020, to March 11, 2020, is taken. In terms of data preprocessing, except for age, which is used as a continuous variable, the other data are classified as categorical variables. The imaging examination results were divided into clearly marked “viral pneumonia,” other lung function categories, and no abnormalities. According to the first hospital’s reference threshold, the results of laboratory tests were classified as “lower/normal/higher” or “normal/higher.” Age was standardized, and other missing elements were filled with 0.

In our study, according to the risk assessment model, patients were divided into three groups: high-risk group, moderate-risk group, and low-risk group. Out of the 2243 patients, 628 (28.00%) were in the low-risk group, 1447 (64.51%) were in the moderate-risk group, and 168 (7.49%) were in the high-risk group. Among them, 17 patients were clinically diagnosed with COVID-19; 16 patients were in the high-risk group, and 1 patient was in the moderate-risk group (see details in Multimedia Appendix 2).

**Figure 7:**
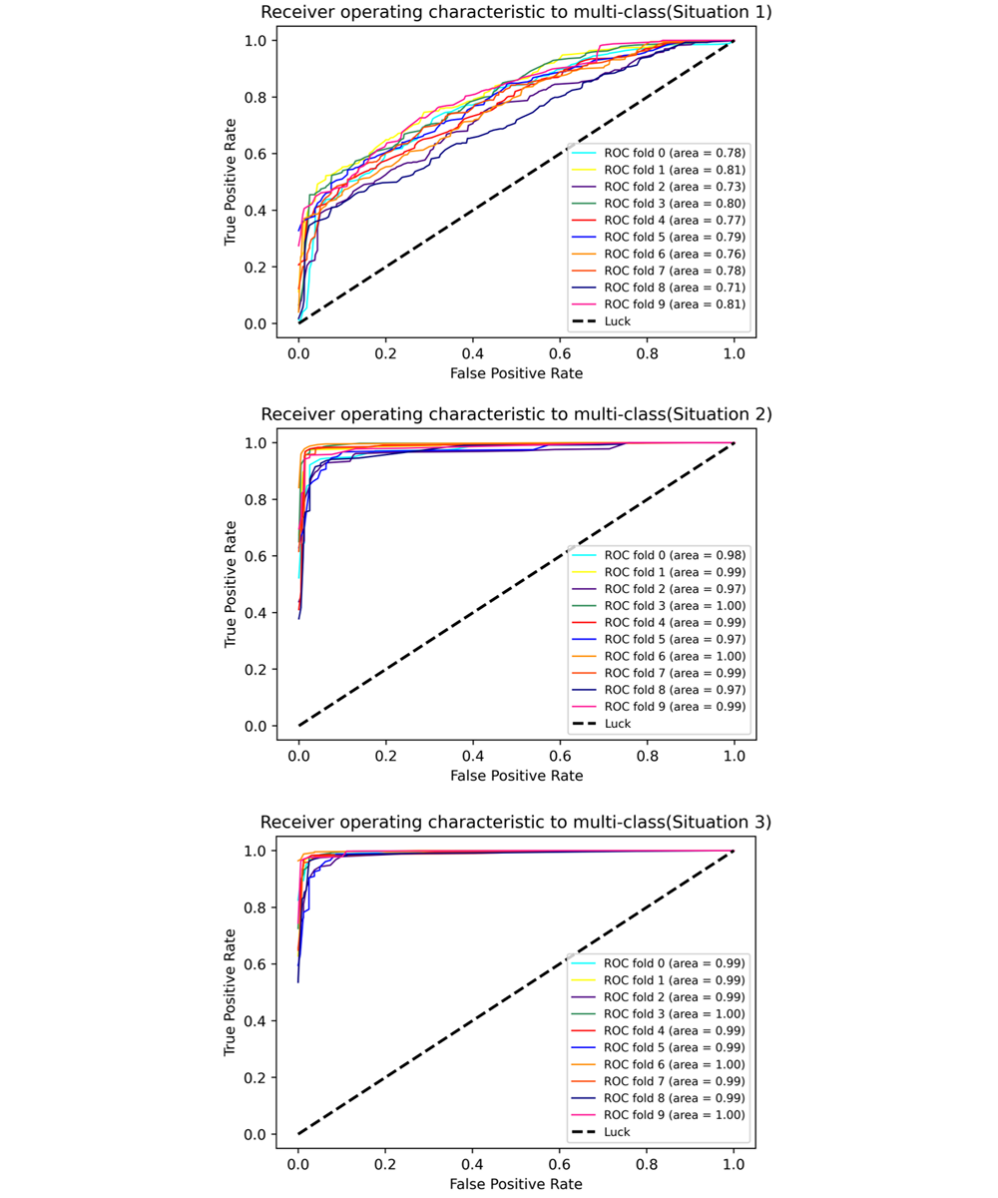
The ROC curve of the dynamic risk stratification model. ROC: receiver operating characteristic.

To ensure the accuracy of risk stratification and to avoid the model overfitting, we used the three categories of low risk, moderate risk, and high risk as labels, and adopted a 10-fold cross-validation method to evaluate and test the model in different scenarios (different dimensions of personal clinical data accessible at the early stage). When we only used the data of patients’ demographic information, clinical symptoms, and contact history, the data set dimensions were (2243, 15). When the results of blood tests were added, the data set dimensions were (2243, 35). After obtaining the CT imaging results of the patient, the data set dimensions were (2243, 36; see details in Multimedia Appendix 2). Figure 7 demonstrates the training set based on the data set in the previously mentioned three scenarios, labels based on the patient's risk level, the receiver operating characteristic (ROC) curve, and its corresponding area under the curve (AUC) value after 10 cross-validation model test results.

As can be seen from the ROC curve and its corresponding AUC value, even if only the information of the patient's epidemiology contact history and clinical symptoms were used, the average value of the three classification results of macro-AUC were all above 0.71. When the data of laboratory tests and imaging were added, the macro-AUC increased to above 0.97. Therefore, the model has a good prediction ability for the above three scenarios. The detailed evaluation indicators of the model are shown in Table 2. All indicators were averaged after 10-fold cross-validation, and Class 1, Class 2, and Class 3 refer to low risk, moderate risk, and high risk, respectively.

**Table 2:** The indicators of the model.

Variables	Situation 1			Situation 2				Situation 3			
	Precision	Recall	F1 score		Precision	Recall	F1 score		Precision	Recall	F1 score
Class 1	0.380	0.576	0.456		0.947	0.956	0.951		0.949	0.956	0.952
Class 2	0.750	0.552	0.634		0.976	0.956	0.966		0.980	0.957	0.968
Class 3	0.750	0.947	0.831		0.841	0.941	0.885		0.850	0.982	0.909
Accuracy	0.588	0.588	0.588		0.955	0.955	0.955		0.959	0.959	0.959
Macroaverage	0.627	0.692	0.640		0.921	0.951	0.934		0.926	0.965	0.943
Weighted average	0.646	0.588	0.599		0.958	0.955	0.956		0.961	0.959	0.959

## Discussion

### Principal Findings

This paper describes a dynamic risk assessment decision support system, which has been used by many Chinese GPs in Zhejiang Province during the COVID-19 outbreak. The DDC19 was designed for GPs working in different situations such as online consultation, assessment evaluation, and triaging in different offline scenarios (community, airport, train station, fever clinic, etc) and following up with suspected patients and discharged patients. It fills in the gap of traditional health care and helps GPs effectively manage residents with different statuses. For patients in DDC19, they can use it to record health data, obtain real time results of risk assessment, and communicate with their GPs without in-person visits; for GPs, they can intuitively grasp their patients’ conditions and provide online advice and interventions in real time. DDC19 contributes to the effective triaging of patients, relieves the pressure of offline clinics of designated hospitals to a certain extent, and reduces cross-infection in the hospital during the COVID-19 outbreak.

With the worldwide spread of COVID-19 and the shortage of medical resources, achieving scientific assessment and effectively triaging the patients in different states is the key to control it. Under the actual clinical condition, GPs need to comprehensively evaluate patients offline without specific symptoms based on their epidemiological contact history, symptoms, laboratory, and imaging findings, and advise in conjunction with the guideline’s recommendations. Although it can assess patients, distinguish them with different risk levels, and find patients who are at high risk, it has a number of inherent defects that cannot meet the need for efficient prevention and control of COVID-19. At present, there is a lack of mobile medical information systems to meet the needs of patient classification, and it is infeasible to truly complete the triage of patients who are potentially infected with SARS-CoV-2 and other patients with similar symptoms before the outpatient clinic of the designated hospital. Researchers at the University of California, San Diego built a number of COVID-19–related tools to support physician’s work based on the EHR system [14]. Although it is beneficial for docking with the process of the original standardized system in the hospital, it cannot directly obtain the data of patients at an early stage and grasp the dynamic conditions of patients. Nowadays, the implementation of early assessment and triage of COVID-19 relies on GP’s knowledge and experience. GPs cannot make a dynamic risk assessment for their patients because they cannot completely grasp the condition of patients at each period in a timely manner. In addition, the problem of a large number of panicked patients with mild symptoms or no risk of COVID-19 going to the designated hospitals during the epidemic has not been resolved.

In the traditional model, clinicians need to obtain patient’s clinical information within a short time by in-person visits. Due to the limitations of time and environment, it may be arduous to assess the patients’ risk for clinicians. In our study, DDC19 can dynamically obtain patients' clinical information by self-report or upload health information, assess their risk level by the dynamic risk stratification logistic regression model, and assist GPs to diagnose and provide recommendations. Therefore, DDC19 helps GPs with online triaging, reduces the pressure of offline clinic, and lowers the risk of cross-infection in the hospital. On the coverage of patient clinical information, our system has been further expanded through questionnaires and inspection reports. The multiclass logistic regression model constructed by the retrospective data (Figure 7 and Table 2) shows that the average recall rate for patients who are high risk reached 0.947, even if the overall accuracy is 0.588 when we only have patients’ contact history and clinical symptoms. As we added the results of blood cell counts and CRP, the accuracy rate of the average classification of the model in different risks reached 0.955, and it is close to 0.959 when considering the results of the lung’s CT. Therefore, our results proved that the dynamic risk stratification model in DDC19 can accurately identify patients who are high risk by their basic health information (epidemiological history, clinical symptoms, and blood tests). DDC19 can help GPs dynamically manage each patient and expand the scope of COVID-19 risk assessment from traditional outpatient clinics to a new type of continuous health care. In addition, the model proves that only considering the contact history data, clinical symptoms, and blood test data available can achieve the risk stratification effect that combines the results of lung CT. It will reduce the burden of the radiologist and unnecessary waste of health resources.

DDC19 is also a new method for using clinical data, which can respond to emergencies conveniently and quickly. The existing COVID-19–related clinical analysis methods focus on establishing new hypotheses or searching for new research evidence. It is difficult to translate into a direct impact on accurate control and rapid response to the epidemic in a short time. Therefore, we built a complete mobile information system that integrates the workflow in the health care system. On the other hand, although there is a lot of research on informationization and AI-related to COVID-19 at the moment [21-24], most of the reported AI-driven tools are still limited to proof-of-concept models. To the best of our knowledge, there is currently only one article about risk assessment and prehospital triaging of patients with suspected COVID-19 [25], but the tools designed by this study are embedded in EHR systems to provide self-triaging and self-scheduling for patients, so doctor-patient interaction and posthospital follow-ups are not supported. In contrast, the mobile information system we designed fits well with the actual triage practice in business processes and supports GPs' real time grasp and assessment of the patient's health status with better flexibility.

### Limitations

This study has some limitations that must be addressed in the next steps of development. First, the complete clinical situation should be considered during the establishment of the dynamic risk assessment model, including the severity of symptoms and history of underlying chronic diseases, but the early retrospective data of fever clinics do not contain these data elements. With further accumulation of relevant clinical data, our system can attempt earlier assessment and triage support methods, and we can also discuss and explore the impact of dynamic changes in patients' clinical information on their COVID-19 risk stratification. Second, as the system is still in the deployment and app stage, the relevant data of patients in the system and in-hospital visits cannot be obtained in a timely manner, so the clinical effects produced by the actual app of the system cannot be evaluated in a timely manner.

### Conclusions

DCC19 is a mobile decision support system designed and developed to assist GPs in providing dynamic risk assessments for potential patients during the COVID-19 outbreak. It collects potential patients’ health information by mobile apps and data transmission mechanisms in different situations, assesses their risk levels through a dynamic risk stratification logistic regression model, and helps GPs manage patients and make further clinical decisions.

## Appendix

Multimedia Appendix 1 The questionnaire of health information.

Multimedia Appendix 2 Statistical distribution of patient data characteristics.

## Abbreviations

AI: artificial intelligence
AUC: area under the curve
COVID-19: coronavirus disease
CRI: credible interval
CRP: C-reactive protein
CT: computed tomography
EHR: electronic health record
GP: general practitioner
NLP: natural language processing
OCR: optical character recognition
ROC: receiver operating characteristic
SARS-CoV-2: severe acute respiratory syndrome coronavirus 2
WHO: World Health Organization
