# Supplementary material for: A COVID-19 Risk Assessment Decision Support System for General Practitioners: Design and Development Study
Source: J Med Internet Res. 2020 Jun 29;22(6):e19786. doi: 10.2196/19786 (PMC7332157; doi:10.2196/19786)
Supplement: Multimedia Appendix 1 [file jmir_v22i6e19786_app1.docx]

Appendix 1：The questionnaire of health information

**The questionnaire of health information**

- **Profile**

*Full Name: （please fill in the blank）

*Date of Birth: please select

*Gender Male Female

*Email:

*Moblie phone: （please fill in the blank）

*Pregnancy No Yes Unknown

- **Questionnaire**

***Q1：Have you ever lived in or been to any affected area in the last 14 days ?**

COVID-19 high incidence areas within the US：New York, New Jersey, Massachusetts, Pennsylvania, California, Illinois, Michigan, Florida, Louisiana, Georgia, Connecticut, Texas, Maryland, Ohio, Washington, Indiana, Colorado, Virginia, Tennessee, North Carolina.

COVID-19 high incidence areas outside the US Western Pacific Region: China, Republic of Korea, Australia, Japan; European Region: Spain，Italy, Germany, France, Switzerland, UK, Russia, Netherlands, Austria, Belgium, Norway, Portugal, Sweden, Turkey, Denmark, Ireland; Eastern Mediterranean Region: Iran, Israel, Saudi Arabia; Region of the Americas: Brazil, Canada, Peru, Chile, Ecuador; South-East Asian Region: India.

Other areas you have travelled or stayed, （please fill in the blank）

No

***Q2：Were there any COVID-19 cases reported in the community where you live in the last 14 days?**

Yes No

***Q3：Have you been in close contact with confirmed or suspected COVID-19 patients in the last 14 days ?**

Yes No

***Q4：Have you been exposed to anyone with fever or respiratory symptoms from COVID-19 high incidence area in the last 14 days ?**

Yes No

***Q5：Do you know any cases of fever or respiratory symptoms in your home, office, school or other crowded places you have been to in the last 14 days ?**

Yes No

- **Symptoms**

***Q6：Have you ever had a fever（body temperature ＞37.3℃/99.1℉）in the last 14 days ?**

Yes please select

Please fill in your highest temperature ℃

No please select

Please fill in your highest temperature ℃

***Q7：Have you experienced any discomfort of the respiratory tract in the last 14 days ?**

Cough

Shortness of breath

Sneezing

Chest tightness

Nasal congestion

Sore throat

Dyspnea

Coughing up blood

Coughing up phlegm please select

Rhinorrhea please select

None

Others，（please fill in the blank）

**Q8：Have you experienced any of the following physical discomforts in the last 14 days ?**

Fatigue

Muscle pain

Diarrhea

Nausea

Vomiting

Headache

Joint pain

Chills

Eye pain

Rash

Palpitation

None

Others，（please fill in the blank）

**Q9：Please fill in your current or recent vital sign results.**

Heart rate:  beats per minute

Blood pressure: mmHg

Respiratory rate: breaths per minute

SpO2:  %

- **Basic Information**

**Q10：Do you have any other comorbidities?**

Hypertension

Diabetes mellitus

Coronary heart disease

Hepatitis B

Chronic bronchopneumonia

Asthma

AIDS

Chronic obstructive pulmonary disease

Chronic kidney disease

Cancer

Cerebral vascular disease

None

Others，（please fill in the blank）

**Q11：Are you allergic to any drug or food?**

None

Some food，（please fill in the blank）

Some drugs，（please fill in the blank）

Others，（please fill in the blank）

- **Lab**

***Q12：COVID-19 Testing?**

Positive

Negative

Pending

Not done

**Q13：COVID-19 specific IgM Antibody Test?**

Positive

Negative

Pending

Not done

**Q14：COVID-19 specific IgG Antibody Test?**

Positive

Negative

Pending

Not done

**Q15：Blood test?**

Normal

Pending

Not done

Abnormal

- **Imaging**

**Q16：Chest CT?**

Normal

Pending

Not done

Abnormal
