# Supplementary material for: A COVID-19 Risk Assessment Decision Support System for General Practitioners: Design and Development Study
Source: J Med Internet Res. 2020 Jun 29;22(6):e19786. doi: 10.2196/19786 (PMC7332157; doi:10.2196/19786)
Supplement: Multimedia Appendix 2 [file jmir_v22i6e19786_app2.docx]

Appendix 1：the details of the Questionnaire

**The questionnaire of health information**

- **Profile**

*Full Name: （please fill in the blank）

*Date of Birth: please select

*Gender Male Female

*Email:

*Moblie phone: （please fill in the blank）

*Pregnancy No Yes Unknown

- **Questionnaire**

***Q1：Have you ever lived in or been to any affected area in the last 14 days ?**

COVID-19 high incidence areas within the US：New York, New Jersey, Massachusetts, Pennsylvania, California, Illinois, Michigan, Florida, Louisiana, Georgia, Connecticut, Texas, Maryland, Ohio, Washington, Indiana, Colorado, Virginia, Tennessee, North Carolina.

COVID-19 high incidence areas outside the US Western Pacific Region: China, Republic of Korea, Australia, Japan; European Region: Spain，Italy, Germany, France, Switzerland, UK, Russia, Netherlands, Austria, Belgium, Norway, Portugal, Sweden, Turkey, Denmark, Ireland; Eastern Mediterranean Region: Iran, Israel, Saudi Arabia; Region of the Americas: Brazil, Canada, Peru, Chile, Ecuador; South-East Asian Region: India.

Other areas you have travelled or stayed, （please fill in the blank）

No

***Q2：Were there any COVID-19 cases reported in the community where you live in the last 14 days?**

Yes No

***Q3：Have you been in close contact with confirmed or suspected COVID-19 patients in the last 14 days ?**

Yes No

***Q4：Have you been exposed to anyone with fever or respiratory symptoms from COVID-19 high incidence area in the last 14 days ?**

Yes No

***Q5：Do you know any cases of fever or respiratory symptoms in your home, office, school or other crowded places you have been to in the last 14 days ?**

Yes No

- **Symptoms**

***Q6：Have you ever had a fever（body temperature ＞37.3℃/99.1℉）in the last 14 days ?**

Yes please select

Please fill in your highest temperature ℃

No please select

Please fill in your highest temperature ℃

***Q7：Have you experienced any discomfort of the respiratory tract in the last 14 days ?**

Cough

Shortness of breath

Sneezing

Chest tightness

Nasal congestion

Sore throat

Dyspnea

Coughing up blood

Coughing up phlegm please select

Rhinorrhea please select

None

Others，（please fill in the blank）

**Q8：Have you experienced any of the following physical discomforts in the last 14 days ?**

Fatigue

Muscle pain

Diarrhea

Nausea

Vomiting

Headache

Joint pain

Chills

Eye pain

Rash

Palpitation

None

Others，（please fill in the blank）

**Q9：Please fill in your current or recent vital sign results.**

Heart rate:  beats per minute

Blood pressure: mmHg

Respiratory rate: breaths per minute

SpO2:  %

- **Basic Information**

**Q10：Do you have any other comorbidities?**

Hypertension

Diabetes mellitus

Coronary heart disease

Hepatitis B

Chronic bronchopneumonia

Asthma

AIDS

Chronic obstructive pulmonary disease

Chronic kidney disease

Cancer

Cerebral vascular disease

None

Others，（please fill in the blank）

**Q11：Are you allergic to any drug or food?**

None

Some food，（please fill in the blank）

Some drugs，（please fill in the blank）

Others，（please fill in the blank）

- **Lab**

***Q12：COVID-19 Testing?**

Positive

Negative

Pending

Not done

**Q13：COVID-19 specific IgM Antibody Test?**

Positive

Negative

Pending

Not done

**Q14：COVID-19 specific IgG Antibody Test?**

Positive

Negative

Pending

Not done

**Q15：Blood test?**

Normal

Pending

Not done

Abnormal

- **Imaging**

**Q16：Chest CT?**

Normal

Pending

Not done

Abnormal

Appendix 2: Statistical distribution of patient data characteristics

Statistical distribution of patient data characteristics

| Feature | Detail | Classified_code | Incidence(%) | Number of records |
| --- | --- | --- | --- | --- |
| Gender | Male | 1 | 47.26 | 1060 |
|  | Female | 0 | 52.74 | 1183 |
| Age | -- | -- | -- | 2243 |
| Epidemiology history | Yes | 1 | 9.72 | 218 |
|  | No | 0 | 90.28 | 2025 |
| Fever | ≤37.2℃ | 0 | 8.69 | 195 |
|  | 37.2℃ - 38.5℃ | 1 | 55.42 | 1243 |
|  | ≥38.5℃ | 2 | 35.89 | 805 |
| Cough | Yes | 1 | 63.35 | 1421 |
|  | No | 0 | 36.65 | 822 |
| Sputum production | Yes | 1 | 25.19 | 565 |
|  | No | 0 | 74.81 | 1678 |
| Fatigue | Yes | 1 | 18.06 | 405 |
|  | No | 0 | 81.94 | 1838 |
| Breathing | Yes | 1 | 4.68 | 105 |
|  | No | 0 | 95.32 | 2138 |
| Chest | Yes | 1 | 5.39 | 121 |
|  | No | 0 | 94.61 | 2122 |
| Pharyngalgia | Yes | 1 | 36.29 | 814 |
|  | No | 0 | 63.71 | 1429 |
| Headache | Yes | 1 | 42.58 | 955 |
|  | No | 0 | 57.42 | 1288 |
| Chills | Yes | 1 | 29.87 | 670 |
|  | No | 0 | 70.13 | 1573 |
| Soreness | Yes | 1 | 32.99 | 740 |
|  | No | 0 | 67.01 | 1503 |
| Stuffy nose | Yes | 1 | 27.78 | 623 |
|  | No | 0 | 72.22 | 1620 |
| Gastrointestinal reactions | Yes | 1 | 10.39 | 233 |
|  | No | 0 | 89.61 | 2010 |
| WBC(10E9/L) | <4.0 | 1 | 7.40 | 166 |
|  | 4.0 – 10.0 | 2 | 63.71 | 1429 |
|  | >10.0 | 3 | 13.33 | 299 |
| GRAN(10E9/L) | <2.0 | 1 | 4.95 | 111 |
|  | 2.0 – 7.0 | 2 | 61.75 | 1385 |
|  | >7.0 | 3 | 17.74 | 398 |
| LYM(10E9/L) | <0.8 | 1 | 19.35 | 434 |
|  | 0.8 – 4.0 | 2 | 64.38 | 1444 |
|  | >4.0 | 3 | 0.71 | 16 |
| RBC(10E12/L) | Male:<4.09,Female:<3.68 | 1 | 5.39 | 121 |
|  | Male:4.09-5.74,  Female:3.68-5.13 | 2 | 76.10 | 1707 |
|  | Male:>5.74,Female:>5.13 | 3 | 2.94 | 66 |
| HGB(g/L) | Male:<131,Female:<113 | 1 | 7.31 | 164 |
|  | Male:131-172,  Female:113-151 | 2 | 74.59 | 1673 |
|  | Male:>131,Female:>113 | 3 | 2.54 | 57 |
| HCT(%) | Male:<38.0,Female:<33.5 | 1 | 6.11 | 137 |
|  | Male:38.0-50.8,  Female:33.5-45.0 | 2 | 76.24 | 1710 |
|  | Male:>50.8,Female:>45.0 | 3 | 2.10 | 47 |
| MCV(fl) | Male:<83.9,Female:<82.6 | 1 | 5.97 | 134 |
|  | Male:83.9-99.1,  Female:82.6-99.1 | 2 | 76.37 | 1713 |
|  | Male:>99.1,Female:>99.1 | 3 | 2.10 | 47 |
| MCH(pg) | Male:<27.8,Female:<26.9 | 1 | 5.22 | 117 |
|  | Male:27.8-33.8,  Female:26.9-33.3 | 2 | 76.95 | 1726 |
|  | Male:>33.8,Female:>33.3 | 3 | 2.27 | 51 |
| MCHC(g/L) | <320 | 1 | 6.06 | 136 |
|  | 320 – 360 | 2 | 77.49 | 1738 |
|  | >360 | 3 | 0.89 | 20 |
| RDW(%) | <11.5 | 1 | 4.28 | 96 |
|  | 11.5 – 14.5 | 2 | 74.68 | 1675 |
|  | >14.5 | 3 | 5.48 | 123 |
| PLT(10E9/L) | Male:<83,Female:<101 | 1 | 1.65 | 37 |
|  | Male:83-303,  Female:101-320 | 2 | 76.73  6.06 | 1721  136 |
|  | Male:>303,Female:>320 | 3 |  |  |
| MPV(fl) | 7.4 – 12.5 | 2 | 82.35 | 1847 |
|  | >12.5 | 3 | 1.43 | 32 |
| PCT(%) | <0.108 | 1 | 1.78 | 40 |
|  | 0.108-0.282 | 2 | 68.08 | 1527 |
|  | >0.282 | 3 | 13.91 | 312 |
| PDW(10(GSD)) | <15.5 | 1 | 75.84 | 1701 |
|  | 15.5-18.1 | 2 | 4.06 | 91 |
|  | >18.1 | 3 | 3.88 | 87 |
| MO(10E9/L) | <0.12 | 1 | 0.49 | 11 |
|  | 0.12-1.00 | 2 | 74.54 | 1672 |
|  | >1.00 | 3 | 9.41 | 211 |
| EO(10E9/L) | <0.02 | 1 | 28.18 | 632 |
|  | 0.02-0.05 | 2 | 55.86 | 1253 |
|  | >0.50 | 3 | 0.40 | 9 |
| BA(10E9/L) | 0.00-0.10 | 2 | 84.31 | 1891 |
|  | >0.10 | 3 | 0.13 | 3 |
| NRBC | -- | Original value | -- | -- |
| IG(%) | 0.0-0.6 | 2 | 75.26 | 1688 |
|  | >0.6 | 3 | 4.50 | 101 |
| CRPH | 0.0-8.0 | 2 | 37.72 | 846 |
|  | >8.0 | 3 | 45.43 | 1019 |
| CT | No obvious abnormalities | 1 | 8.25 | 185 |
|  | Other imaging manifestations | 2 | 13.02 | 292 |
|  | Viral pneumonia manifestations | 3 | 0.76 | 17 |
